# Essential oil components of turmeric inhibit hepatic lipidification and liver fibrosis in a diet-induced NASH model rats

**DOI:** 10.1038/s41598-023-47097-6

**Published:** 2023-11-25

**Authors:** Yukari Watanabe, Hitoshi Watanabe, Sarasa Kogure, Yuri Tanioka, Jun Yamauchi, Tadasu Furusho

**Affiliations:** 1 Department of Ecological Symbiotic Science, Graduate School of Agriculture, Tokyo University of Agriculture, 1-1-1 Sakuragaoka, Setagaya, Tokyo 156-8502 Japan; 2 Department of International Food and Agricultural Science, Faculty of International Agriculture and Food Studies, Graduate School of Agriculture, Tokyo University of Agriculture, 1-1-1 Sakuragaoka, Setagaya, Tokyo 156-8502 Japan; 3 Department of International Food and Agricultural Science, Faculty of International Agriculture and Food Studies, Tokyo University of Agriculture, 1-1-1 Sakuragaoka, Setagaya, Tokyo 156-8502 Japan

**Keywords:** Chemical biology, Diseases, Health care

## Abstract

In this study, the fraction extracted from turmeric powder with 50% ethanol and fractionated with n-hexane were administered to diet-induced NASH model rats. NASH model was prepared with SD rats by feeding an originally designed choline-deficient, high-fat, high-fructose (HFF-CD) diet for 10 weeks. To the HFF-CD diet, hexane fraction and 50% ethanol fraction after hexane fractionation were added at 100 mg/kg body weight. 10 weeks later, blood samples and liver were collected for the following parameters: lipid weights, serum ALT, AST, TG, liver TG, TBARS levels, lipid metabolism-related gene expression and histopathological examination of the liver. As the results, the hexane fraction and 50% ethanol fraction showed a decrease in lipid weight, a decrease in hepatic TG, and activation of PPAR-α in the lipid metabolism-related gene test. These results suggest that the hexane fraction of turmeric has an inhibitory effect on fat accumulation in the liver by promoting lipid metabolism in NASH model rats.

## Introduction

In recent years, the number of patients with lifestyle-related diseases such as obesity, diabetes, and hypertension has been on the increase in Japan due to the westernization of food and changes in lifestyle. In addition, due to the increased consumption of soft drinks containing fructose/glucose, non-alcoholic fatty liver disease (NAFLD) is becoming the most common liver disease in the twenty-first century. It is estimated that 10–20% of these patients will develop non-alcoholic steatohepatitis (NASH). In Japan the prevalence of NAFLD ranges from 9 to 30%, with an estimated 1 million people in Japan with NASH, given that the prevalence of NASH is 3–5% worldwide^1^. Of concern is that the estimated prevalence of NAFLD worldwide has increased from 20.3% in 2000–2005 to 26.8% in 2011–2015, suggesting that the prevalence of NASH is likely increasing in parallel with the increase in NAFLD^2^. NASH, which progresses from cirrhosis to hepatocarcinoma, is a liver disease gaining attention in Japan, but no treatment has yet been established. The pathogenesis of NASH is generally understood based on the two-hit theory^3^. The first hit is fatty liver due to accumulation of neutral fat in the liver caused by lifestyle-related diseases such as obesity, diabetes, and hypertension, and the second hit is NASH due to inflammatory cytokines and oxidative stress. More recently, a more complex multiple parallel hits hypothesis has been proposed, in which disturbances in the intestinal microflora and the effects of numerous factors released from the intestinal tract and adipose tissue contribute to the pathophysiology of the disorder^4^.

Turmeric is a perennial herb of the ginger family native to tropical Asia, and includes Curcuma longa (autumn turmeric), C. aromatica (spring turmeric), and C. zedoaria (purple turmeric), and its rhizomes have been used since ancient times as a food ingredient, dye, and traditional medicine. Recently, many health foods in Japan have been supplemented with C. longa in an effort to maintain or improve liver function. C.longa contains 1–5% polyphenolic curcuminoids and about 5% other essential oil components. Curcuminoids include curcumin, demethoxycurcumin, and bisdemethoxycurcumin. Curcumin is known to have antioxidant^5^, antitumor^6^, anti-inflammatory^7^, and hypoglycemic^8^ effects, while its essential oil components are reported to have anti-inflammatory effects^9^ and to improve blood flow^10^. However, the effects of turmeric essential oil on NASH are not known.

In this study, the n-hexane and 50% ethanol fractions fractionated from turmeric powder were administered and their effects on liver function were examined.

There are established animal models of NASH, including genetic models and diet-induced models created by ingestion of choline-deficient amino acid diets (CDAA diet) or methionine-choline-deficient diets (MCD diet) in mice and rats^11–13^. However, some models present fatty liver but not inflammation or fibrosis, while some models present fatty liver, inflammation, and fibrosis but exhibit phenotypes opposite to human NASH.

In this study, we attempted to generate a diet-induced NASH rat model by using our original choline-deficient high-fat, high-fructose diet (HFF-CD diet).

## Results

### Components in 50% ethanol fraction and hexane fraction

**Figure 1:**
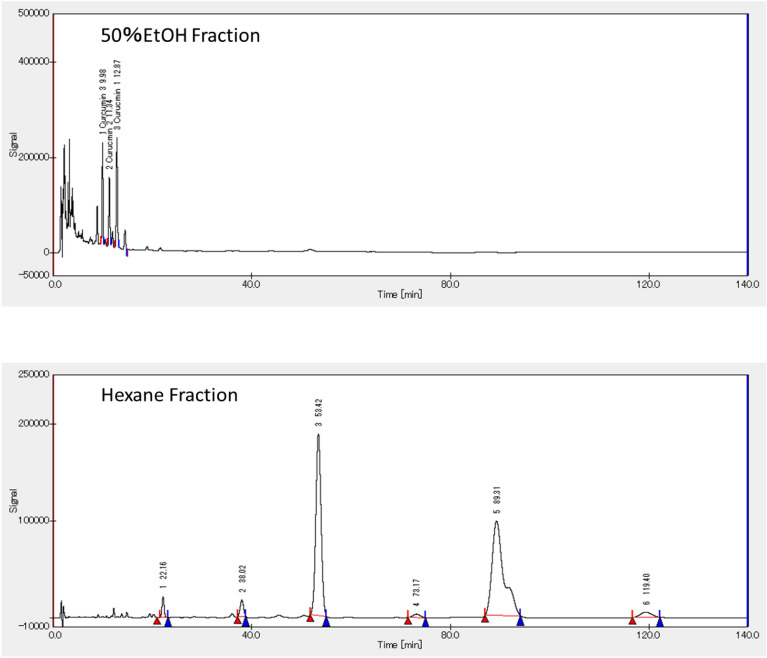
Chromatogram of 50% ethanol fraction and hexane fraction.

Chromatograms of 50% ethanol fraction and hexane fraction are shown in Fig. 1. Three types of curcuminoid peaks were confirmed in the 50% ethanol fraction: curcumin at 9.98 min, curcumin 2 at 11.34 min, and curcumin 3 at 12.87 min. On the other hand, no curcuminoid peaks were observed in the hexane fraction, and large peaks were detected at 53.42 min and 89.31 min. These two peaks were fractionated by column chromatography and eluted with n-hexane: ethyl acetate (99:1). As a result of analysis using LC–MS/MS, the peak at 53.42 min was identified as ar-turmerone (MW = 215) and the peak at 89.31 min as β-turmerone (MW = 218).

### Change in body weight

**Figure 2:**
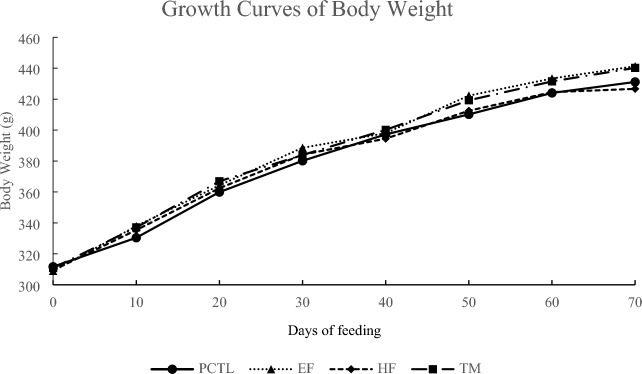
Effects on growth curves of body weight in Nash model rats.

Changes in body weight in each group are shown in Fig. 2. The EF group, HF group, and TM group all showed almost the same body weight change as the PCTL group, and there was no significant difference between groups.

### Liver and adipose tissue weight

Liver weight and adipose tissue weights per Kg body weight are shown in Table 1. Liver weights were almost the same in the HF, EF and TM groups as in the PCTL group.

**Table 1:** Effects on weights of liver, perirenal fat, epididymal fat, peritoneal fat, and brown fat in NASH model.

Group		PTCL	EF	HF	TM
Liver weight	(g/Kg. BW)	59.9 ± 1.5	59.4 ± 1.1	61.4 ± 1.4	60.5 ± 1.4
Perirenal fat	(g/Kg. BW)	25.9 ± 1.0	19.6 ± 1.4**	20.5 ± 1.4*	20.4 ± 0.8*
Epididymal fat	(g/Kg. BW)	20.9 ± 1.0	16.9 ± 0.7**	15.6 ± 0.2**	16.6 ± 0.8**
Peritoneal fat	(g/Kg. BW)	9.8 ± 1.0	7.7 ± 0.5	7.2 ± 0.6	7.6 ± 0.8
Brown fat	(g/Kg. BW)	1.1 ± 0.1	1.6 ± 0.1	1.2 ± 0.2	1.1 ± 0.1

Each value shows mean ± S.E. Significantly different from the PTCL group (*p < 0.05, **p < 0.01 by Dunnett’s test).

In perirenal fat weight, there was significant decrease in the HF, EF and TM groups compared to the PCTL group. A similar significant decrease (p < 0.01 or 0.05) were also observed in epididymal fat weight (p < 0.01). In peritoneal fat weight, there were no significant differences in the EF, HF and TM groups compared with the PCTL group. However, they showed a trend towards lower values.

In contrast, no significant differences were observed in brown fat weight between the four groups.

### Blood chemical analysis

The results of blood chemical analysis in the serum are shown in Table 2. ALT levels were significantly lower in the EF and TM groups than in the PCTL group. AST levels were significantly lower in the EF group than in the PCTL group. In the HF group, ALT and AST tended to be lower than in the PCTL group, although there was no significant difference. The TG levels were significantly lower in the HF group than in the PCTL group.

**Table 2:** Effects on blood chemical in serum and liver of NASH model rats.

Group		PTCL	EF	HF	TM
AST	(IU/L)	186.7 ± 20.0	127.9 ± 7.8*	152.6 ± 13.0	142.7 ± 8.2
ALT	(IU/L)	102.1 ± 12.3	61.7 ± 4.6*	81.1 ± 13.7	61.4 ± 5.7*
Serum TG	(mg/dL)	54.8 ± 3.4	47.0 ± 5.8	37.7 ± 3.3*	49.6 ± 1.7
Hepatic TG	(g/Total liver)	1.77 ± 0.19	1.55 ± 0.13	1.06 ± 0.13*	1.57 ± 0.20
Hepatic TBARS	(mmol/g/protein liver)	878.3 ± 78.6	711.6 ± 57.5	726.2 ± 14.6	554.5 ± 43.1**

Each value shows mean ± S.E. Significantly different from the PTCL group (*p < 0.05, **p < 0.01 by Dunnett’s test).

### Hepatic TG and TBARS levels

Hepatic TG and TBARS levels are shown in Table 2. Hepatic TG levels were significantly lower in the HF group than in the PCTL group. There was no significant difference between the EH group and the TM group compared to the PCTL group, but the level tended to be lower than that of the PCTL group. Hepatic TBARS levels tended to be lower in the EF and HF groups than in the PCTL group, but there was no significant difference. However, TBARS levels in the TM group were significantly lower than those in the PCTL group.

### Relative expression levels of lipid metabolism-related genes

**Figure 3:**
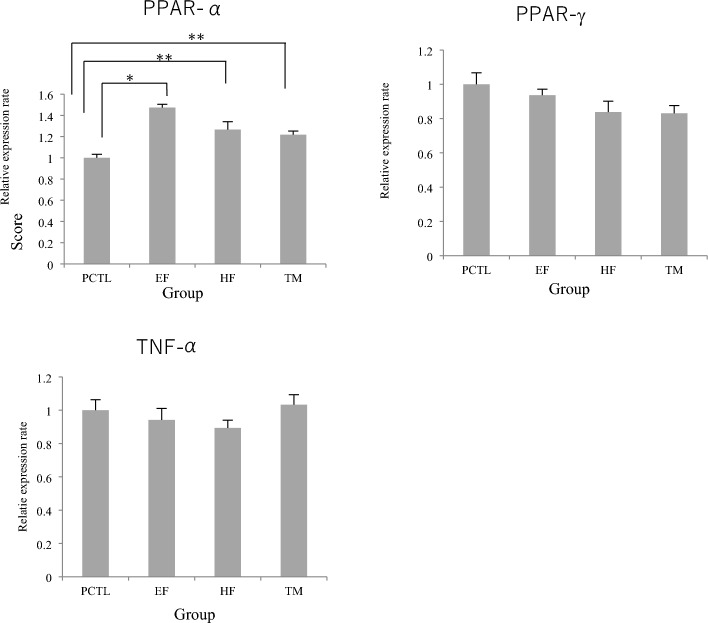
Effects on expression levels of PPAR-α, PPAR-γ and TNF-α in Nash model rats. Bars represent mean ± S.E. Significantly different from the PCTL group (**p < 0.01 by Dunnett’s test).

The results of measuring the relative expression levels of genes related to lipid metabolism are shown in Fig. 3. PPAR-α expression levels in the HF, EF and TM groups were significantly higher than those in the PCTL group. PPAR-γ expression levels were low in all groups, but no significant difference from the PCTL group was seen. In addition, although TNF-α expression levels in the 3 groups was lower than that in the PCTL group, no significant difference was seen.

### Macroscopic and histopathological examination

**Figure 4:**
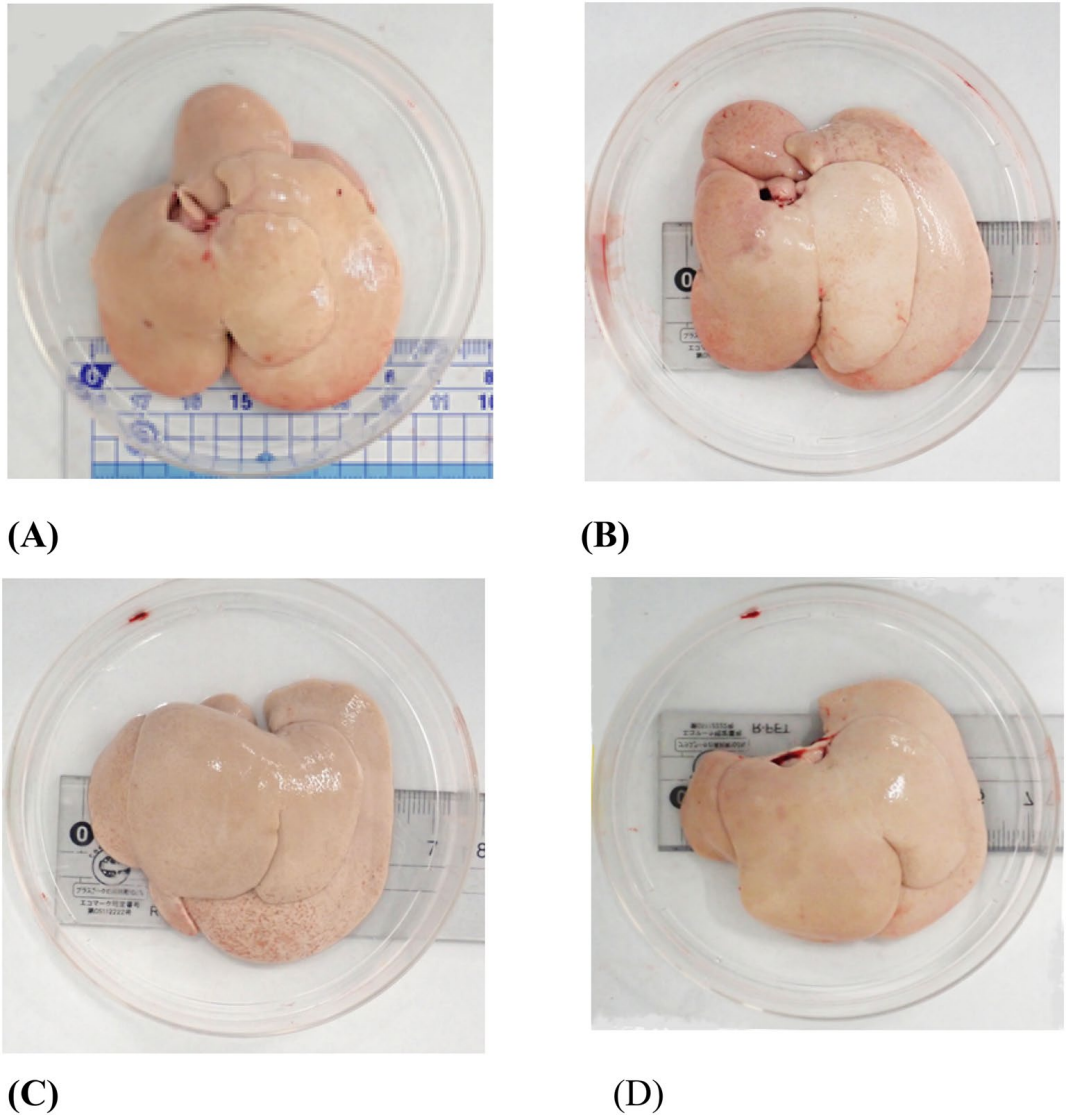
Macroscopic photos of liver in Nash model rats. (**A**) PCTL, (**B**) EF, (**C**) HF, (**D**) TM.

**Figure 5:**
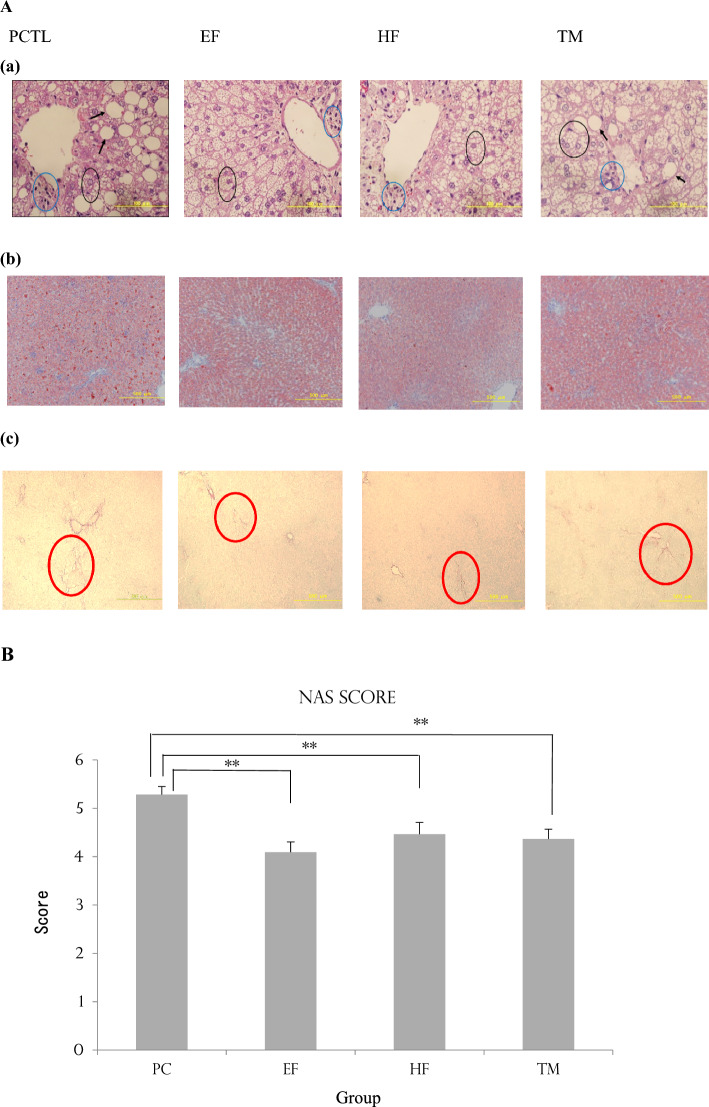
Histological examination of liver in NASH model rats and NAFLD activity score (NAS). (**A**) Histological examination of liver in NASH model rats. (**a**) HE stain, (**b**) Oil Red O stain, (**c**) Sirius Red Stain. (**a**) Black arrows indicate large vacuoles. Blue circles indicate inflammation hepatocyte. Black circles indicate ballooning hepatocyte. (**c**) Red circles indicate area of fibrosis. (**B**) NAFLD activity score (NAS). Significantly different from the PTCL group (**p < 0.01 by Dunnett’s test).

Macroscopic photos of liver are shown in Fig. 4, and the HE-stained image, the Oil Red O-stained image and the Sirius Red-stained image are shown in Fig. 5A. NAFLD activity score (NAS) is shown in Fig. 5B.

Macroscopocally, the livers of rats became white and fatty in all groups.

On HE-staining, the PCTL group was interspersed with areas of hepatocytes with large vacuoles. In addition, hepatocytes swollen due to the filling of small vacuoles accounted for the majority. The EF and HF groups were mostly hepatocytes with small vacuoles. In the TM group, areas of hepatocytes with large vacuoles were scattered, similar to the PCTL group.

Oil Red O staining was strongly positive in the entire region of the PCTL and TM groups. Although the EF and HF groups showed strong positive results, weakly positive areas were observed among them, suggesting that fat deposition was suppressed.

Sirius red staining showed no difference in the degree of fibrosis in the EF, HF and TM groups compared to the PCTL group, ranging from very mild to mild.

The PTCL group scored more than 5 points and was classified as NASH. The EF, HF, and TM groups scored in the 4-point range and were in the borderline region, with significantly lower scores relative to the PTCL group.

## Discussion

NASH is considered to be strongly associated with obesity, insulin resistance, type 2 diabetes, and dyslipidemia (i.e. metabolic syndrome) and is considered the hepatic manifestation of these diseases^14^. In particular, hepatocellular carcinoma and cirrhosis are the leading causes of death among diabetic patients, suggesting that NASH may be responsible for these deaths, with liver disease being a major contributor^15^. Currently, NASH is the most common liver disease in the world. It can progress to cirrhosis and hepatocellular carcinoma making it desirable to establish preventive and therapeutic measures as soon as possible. Analysis of the pathogenesis of NASH using mice and rats as animal models is an important step in understanding NASH. Developing appropriate animal models of NASH is essential. A number of genetic and diet-inducible animal models have been reported^16^. However, there are many cases in which models present fatty liver but not inflammation or fibrosis. Others, such as the methionine-choline deficient diet model, exhibit fatty liver, inflammation, fibrosis, and hepatocarcinogenesis, while others exhibit phenotypes opposite to those of human NASH, such as weight loss and hypoglycemia. In this study, we attempted to clarify the effects of turmeric essential oil components on NASH models, while at the same time modifying the diet composition of previously reported diet-inducible models and feeding them to SD rats to induce NASH. Therefore, a choline-free diet was prepared by adding an amino acid mixture as a protein source under conditions of high fat, high fructose, and high cholesterol (HFF-CD diet). The histopathological examination of the livers of rats fed on this diet for 10 weeks, revealed large vacuoles, inflammation hepatocyte and ballooning hepatocyte (HE staining) and liver fattening (Oil Red O staining) in PTCL group. Fibrosis was also confirmed by Sirius Red staining. These results may reflect the enlarged liver and white in color due to fatty liver at necropsy, as well as increased total lipid and triglyceride levels in the liver on biochemical examination. Applying the histopathology results to the NAS score resulted in a score of 5 or more, which is judged to be NASH. These results indicated that our diet composition is capable of inducing NASH in SD rats. Therefore, we investigated the effects of essential oil components extracted from turmeric on hepatic lipogenesis and fibrosis by feeding SD rats with NASH induced by HFF-CD diet.

The 50% ethanol extract of turmeric powder was fractionated to prepare a hexane fraction (HF) and a 50% ethanol fraction of the residue of the hexane fraction (EF). The prepared fractions were analyzed by LC–MS and confirmed to contain curcuminoids in the EF and ar-turmerone and β-turmerone, sesquiterpenoids, in the hexane fraction. EF, HF and turmeric powder (TM) were added to HFF-CD diet and fed to rats.

As a result, there was a significant (P < 0.01 or 0.05) decrease in the weight of epididymal and perirenal fat in the EF, HF and TM group compared to the PCTL group. Peritoneal fat weight was also significantly (P < 0.05) decreased in the EF and HF groups compared to the PCTL group. On the other hand, there were no differences in brown fat weight between all groups. Brown fat is reported to be involved in body fat regulation, and we believe that the increase in lipid-based heat production occurred, resulting in the reduction of other body fat. Since brown fat activating capacity has been confirmed for many food components such as capsicum, garlic, and ginger^17^, it is possible that the components in turmeric also directly activate brown fat.

Serum TG was significantly lower in the HF group than in the PCTL group and was also lower in the other two groups. Hepatic TG was significantly (p < 0.05) lower in the HF group than in the PCTL group, and the other two groups also showed a decreasing trend. The reason why TG in serum and liver was reduced by the administration of turmeric extract may be changes in the expression of genes related to lipid metabolism.

PPAR-α expression was significantly (P < 0.01) increased in three groups compared to the PCTL group. PPAR-γ expression tended to be decreased in all groups compared to the PCTL group.

The results of HE staining and Oil Red O staining showed a reduction of TG in the HF and EF groups with an inhibition of the increase of fat droplets in the liver. These results indicate that the hexane and 50% ethanol fractions of turmeric inhibited hepatic lipidification in a diet-induced NASH rat model. This suggests that PPAR-α is activated by HF and EF, promoting β-oxidation of fatty acids and reducing liver TG. PPAR-α has been reported to decrease serum TG and TBARS^18^ and in this study, a significant decrease in serum TG and liver TBARS were also observed. On the other hand, a decreasing trend in PPAR-γ expression was observed. In a study using liver-specific PPAR-γ-deficient ob/ob mice, it was reported that PPAR-γ deficiency dramatically improved fatty liver with a decrease in lipid droplets, and that PPAR-γ is closely involved in fatty liver development^19^. This suggests that the suppression of hepatic lipid droplets in the HF and EF groups in this study was also influenced to a small extent by PPAR-γ reduction. In addition, turmeric powder was significantly more effective in inhibiting oxidative stress, although it had a smaller effect in inhibiting liver lipidisation. This was presumably due to the strong effect of the antioxidant properties of curcuminoids, which are abundant in turmeric.

Regarding liver fibrosis, the results of Sirius red staining showed a slight suppression of fibrosis in the HF and EF groups. This was thought to be due to a tendency to suppress the expression of TNF-α, a hepatitis marker, as the inflammatory cytokine TNF-α was reduced in the EF and HF groups, although there were no significant differences. Furthermore, ROS production, an indicator of oxidative stress in the liver, was measured by the TBA method^20^, the EF and HF groups showed lower values although not significantly and the inflammatory cytokine TNF-α is decreased in the EF and HF groups, although not significantly different from the PCTL group. This was presumably due to the suppression of lipidification and oxidative stress in liver parenchymal cells by the administration of turmeric extract.

**Figure 6:**
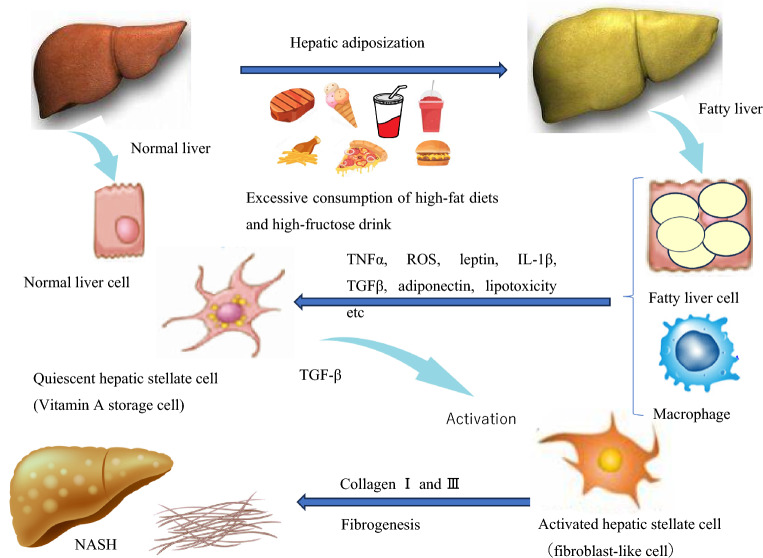
Excessive consumption of high-fat diet and high-fructose drinks in relation to hepatic steatosis and liver fibrosis and NASH.

Activation of hepatic stellate cells plays a major role in liver fibrosis in many liver diseases, including NASH (Fig. 6). Excessive consumption of high-fat diets and high-fructose beverages is known to exacerbate type 2 diabetes^21^ as well as increase hepatic lipidisation. Increased ROS production, increased oxidative stress due to lipotoxicity and excessive release of the inflammatory cytokine TNF-α are induced in fatty hepatocytes. At the same time, macrophages produce inflammatory cytokines such as TGF-β, TNF-α and IL-1β. These actions have been reported to activate hepatic astrocytes, which are responsible for vitamin A storage during quiescence, leading to their transformation into fibroblast-like cells and the release of large amounts of collagen, which in turn lead to liver fibrosis^22^. Therefore, the intake of ingredients with anti-inflammatory, antioxidant and β-oxidation-induced lipolysis may be an effective means to inhibit the progression of NASH caused by excessive high-fat, high-fructose intake.

It has been reported that turmeric intake improves lipid metabolism in rats fed a high-fructose diet^23,24^. Antihyperlipidemic effects have also been reported for turmeric oil^25^. Lizbeth et al. reported that administration of curcumin to db/db mice, a model of diabetes, increased AMPK activity and enhanced fatty acid oxidation, thereby suppressing hepatic lipidification^26^. Furthermore, it has been reported that sesquiterpenes in the herb Cyclolepis genistoides D. Don (palo azul), which is native to Paraguay, increase AMPK activity and promote fatty acid oxidation^27^. Since the hexane and 50% ethanol fractions in turmeric contain sesquiterpenoids and curcuminoids, the mechanism by which the hexane and 50% ethanol fractions in turmeric enhance lipid metabolism may not only be due to elevated PPAR-α and decreased PPAR-γ, but may also involve enhanced fatty acid oxidation through increased AMPK activity.

In this study, it was suggested that essential oil components of turmeric have the ability to reduce hepatic lipidification and fibrosis in NASH model rats, but the mechanism by which the extracted components act needs to be clarified in more detail in the future.

## Materials and methods

### Experimental materials

#### Preparation of 50% ethanol fraction and hexane fraction

Indian C. longa powder was purchased (Ecology Health Lab Co., Ltd., Japan), and samples were prepared according to the following procedure. Five liters of 50% ethanol was added to 100 g of turmeric powder and extracted with stirring at room temperature for 48 h. Three hundred milliliters of the 50% ethanol extract after suction filtration was transferred to a separating funnel, the same amount of n-hexane was added and stirred, and after phase separation, the lower 50% ethanol layer was recovered. This procedure was repeated twice. The 50% ethanol layer was directly concentrated (Ethanol fraction: EF), and the n-hexane layer was dehydrated with anhydrous sodium sulfate and concentrated (Hexane fraction: HF).

#### HPLC analysis

The EF and HF were analyzed using a high performance liquid chromatograph (HPLC). As curcuminoid standards, curcumin (Fujifilm Wako Pure Chemical Industries, Ltd.), curcumin 2 (demethoxycurcumin) and 3, (bis-demethoxycurcumin) (Nagara Science) were used. HPLC was performed using a pump: LC-10AD (Shimadzu Corporation), autosampler: SIL-20A (Shimadzu Corporation), column oven: CTO-10A (Shimadzu Corporation), UV–visible detector: SPD-10A (Shimadzu Corporation), data processing software: Chromato-Pro (Run Time Corporation) at an absorbance 240 nm. Separation was performed using Inertsil ODS-3 (Φ4.6 × 250 mm, GL Sciences), column temperature: 40 °C, mobile phase: water/acetonitrile (40 /60), flow rate: 0.75 mL/min.

#### Fractionation of hexane fraction by column chromatography

A glass chromatography column (Φ20 × 300 mm) was filled to 15 cm with silica gel for column chromatography (C-200, Fuji Film Wako Pure Chemical Industries, Ltd.) wetted with n-hexane. One gram of HF fraction dissolved in 2.0 ml of n-hexane, n-hexane: 200 mL → n-hexane: ethyl acetate (9:1): 200 mL → n-hexane: ethyl acetate (1:1): 200 mL → n-hexane: ethanol (99:1): 200 mL → n-hexane: ethanol (1:1): 200 mL → ethanol 200 mL → chloroform 200 mL → methanol: 200 mL developed in that order. The fractionated eluate was concentrated under reduced pressure and analyzed qualitatively by HPLC.

#### Analysis by LC–MS/MS

The n-hexane: ethyl acetate (99:1) fraction was analyzed for sesquiterpenoids using LC–MS/MS. As sesquiterpenoid standards, Bisacrone, ar-Turmerone, α-Turmerone, Turmeronol A, and Turmeronol B (Nagara Science) were used. For LC–MS/MS, pump: LC-20AD (Shimadzu Corporation), autosampler: SIL-20A (Shimadzu Corporation), column oven: CTO-20A (Shimadzu Corporation), mass spectrometer: LCMS-8040 (Shimadzu Corporation), data processing software: Lab Solution (Shimadzu Corporation), TSK gel ODS-100 V 3 μm (Φ2.0 × 150 mm, Tosoh) (for separation), column temperature: 40 ℃, mobile phase: acetonitrile containing 0.1% formic acid: water (60/40), flow rate: 0.2 mL/min. The nebulizing gas flow rate was 3 L/min, the drinking gas flow rate was 15 L/min, the desolvation line temperature was 250 °C, and the heat block temperature was 400 ℃.

### Effects of turmeric extract administration on NASH model rats

#### Diet

The diet composition for producing diet-induced NASH animals is shown in Table 3. An originally designed choline-deficient high-fat, high-fructose diet (HFF-CD) was prepared by modifying the AIN76 diet composition. For the EF and HF diets, EF or HF was added to the HFF-CD diet at 100 mg/kg body weight. A TM diet was prepared by adding 3% turmeric powder to the HFF-CD diet.

**Table 3:** Composition of diet (% w/w).

Items	PCTL	EF	HF	TM
Amino acid mixture	20	20	20	20
DL-methionine	0.3	0.3	0.3	0.3
Powdered fat (lard)*	38.5	38.5	38.5	38.5
Powdered fat (canola oil)*	7.2	7.2	7.2	7.2
Striped corn oil**	0.5	0.5	0.5	0.5
Fructose	22.5	22.5	22.5	22.5
Vitamin mixture	1.0	1.0	1.0	1.0
Mineral mixture	3.5	3.5	3.5	3.5
Cellulose powder	5.0	5.0	2.0	5.0
Sodium cholate	0.5	0.5	0.5	0.5
Cholesterol	1.0	1.0	1.0	1.0
50% ethanol extract fraction	–	10 mg/Kg・BW	–	–
Hexane extract fraction	–	–	10 mg/Kg・BW	–
Turmeric powder	–	–	–	3.0
Total	100	100	100	100

*Each powdered fat contained approximately 65% fat and 35% maltodextrin.

**Stripped corn oil contained vitamins A, E, and D according to the AIN76 diet composition.

### Experimental animals

For the experiment, 9-week-old, male, Sprague–Dawley rats (Japan SLC) were obtained. After 7 days of acclimation, rats were divided into 4 groups (n = 5) so that each group had approximately the same average body weight. The control (PCTL), EF, HF, and TM groups were fed with HFF-CD diet, EF diet, HF diet and TM diet, respectively. The animals were fed with pair feeding of 13 g/day. The body weights were recorded once a week. The animals were kept in a clean breeding room with the temperature maintained at 23℃, and the humidity held at 60% to 65% on a 12-h light and dark cycle, and tap water was freely given as drinking water.

All rats were euthanized 10 weeks after the start of the test feeding. Rats were placed in a laboratory animal anesthesia machine (SN-487-OT, Shinano Manufacturing Co., Ltd., Japan) and anesthetized with isoflurane by inhalation. The rats were then laparotomized and euthanized by whole blood collection from the inferior vena cava, and then the liver, brown fat, perirenal fat, peritoneal fat and epididymal fat were collected and weighed.

A portion of the collected liver was divided for histopathological examination and fixed in 10% neutral formalin (Mildform 10N, Fujifilm Wako Pure Chemical Industries, Ltd.). The remaining divided liver was stored frozen.

### Blood chemical analysis

The collected blood was placed in a serum separation tube (Separapid Tube S, Sansho) and centrifuged at 2,500 rpm for 15 min to obtain serum. Aspartate aminotransferase (AST), Alanine amino Transferase (ALT) and Triglyceride (TG) were measured.

### Liver triglyceride (TG) and lipid peroxides (TBARS) measurement

TG in the liver was measured according to the Folch method^28^. After homogenizing the cryopreserved liver with methanol, chloroform was added at a ratio of 2:1 and mixed at 40 °C for 1 h. After standing overnight, it was centrifuged for 15 min at 3000 rpm. The chloroform layer was separated, dehydrated with anhydrous sodium sulfate, filtered, and the filtered extract was concentrated and dried. The residue was dissolved in 2-propanol, and used as a sample. TG was measured using Triglyceride E Test Wako (Fuji Film Wako Pure Chemical Industries, Ltd.). The amount of TBARS in the liver was determined according to the TBA method from the cryopreserved liver following the method of Ohkawa et al.^20^. The cryopreserved liver was homogenized with 1.15% potassium chloride solution, and then heated in a boiling water bath at 95 °C or higher for 60 min. After cooling, n-butyl alcohol: pyridine (5:1) was added, and the mixture was extracted with stirring, centrifuged, and the upper layer was used as a sample. The amount of TBARS was expressed as the amount per 1 g of liver protein determined by the Lowry method.

### Relative expression levels of lipid metabolism-related genes

Gene expression levels of PPAR-α, PPAR-γ and TNF-α, factors related to lipid metabolism in the liver, were determined by RT-qPCR. rRNA extraction was performed using Spasol®-RNA I SuperG (Nakarai Tesque). cDNA was prepared from a constant volume of 400 ng of the extracted RNA and PPAR-α, PPAR-γ and TNF-α expression levels were measured by RT-PCR using the primers shown below.

PPAR-α primers were GGACAAGGCCTCAGGATACCACTA (forward) and GACATCCCGACGGACAGGCACT (revers), PPAR-γ primers were CCTCCCTGATGAATAAAGATGG (forward) and CACAGCAAACTCAAACTTAGGC (revers), TNF-α primers were TTCCGAATTCACTGGAGCCTCGAA (forward) and TGCACCTCAGGGAAG.

AATCTGGAA (revers), GCCATGCATGTCTAAGTACGC (forward) and CCGTCGGCATGTATTA.

GCTC (revers) were used as primers for 18S rRNA. THUNDERBIRD Next SYBR qPCR Mix (Toyobo) and CFX Connect Real-Time System (Bio-Rad) were used for amplification and quantification in the PCR method. The gene expression level was corrected with the expression level of 18S rRNA by the internal standard method.

### Liver histopathological examination

After embedding the liver piece fixed with Mildform 10N in paraffin, a thin section was prepared. Paraffin sections were stained by Hematoxyline-Eosin (HE) staining, Oil Red O staining and Sirius Red-Fast Green staining (Sirius red staining). Fat accumulation in the liver was observed by Oil Red O staining, and fibrosis by Sirius Red staining.

### Ethical statement

Animal experiments were conducted with the ethical approval of the Tokyo University of Agriculture Experimental Animal Committee (approval number 2021086). In addition, it was conducted in accordance with the Prime Minister's Office's "Laboratory Animal Management and Storage Standards", the Tokyo University of Agriculture's "Laboratory Animal Management Guidelines" and the ARRIVE guidelines.

### Statistical analysis

The experimental results were statistically analyzed using statistical analysis software "STATCEL Ver.4", and the average value and standard error for each group were calculated. Dunnett’s test was performed for the significance test. The significance level was defined as a risk rate of less than 5% and was shown as less than 5% and less than 1%.

## Data Availability

The datasets used during the current study available from corresponding author on reasonable request.
